# Acquired factor V inhibitor in a case of pediatric venous thrombosis

**DOI:** 10.1016/j.rpth.2024.102646

**Published:** 2024-11-29

**Authors:** Sweta Gupta, Matthew W. Bunce, Emily A. Cid, Rodney M. Camire, Amy D. Shapiro

**Affiliations:** 1 Department of Pediatric Hematology, Innovative Hematology, Indiana Hemophilia and Thrombosis Center, Indianapolis, Indiana, USA; 2 Division of Hematology and the Raymond G. Perelman Center for Cellular and Molecular Therapeutics, The Children’s Hospital of Philadelphia, Philadelphia, Pennsylvania, USA; 3 Department of Pediatrics, Perelman School of Medicine, University of Pennsylvania, Philadelphia, Pennsylvania, USA

**Keywords:** acquired FV inhibitor, anticoagulation, direct oral anticoagulants, factor V activity, thrombosis

## Abstract

**Background:**

The development of acquired factor (F)V with inhibitor (AFVwI) is rare, resulting mainly in bleeding complications, although sporadic cases of thrombosis in adults have been reported.

**Key Clinical Question:**

How do you diagnose and manage a pediatric case of acute deep venous thrombosis associated with the concurrent finding of AFVwI?

**Clinical Approach:**

A 13-year-old female with Crohn's Disease and May–Thurner anatomy developed extensive deep venous thrombosis of the left lower extremity, complicated by the finding of AFVwI, discovered during the evaluation of a prolonged prothrombin time and a low FV activity. Anticoagulation was initiated with low-molecular-weight heparin followed by a direct oral anticoagulant, rivaroxaban, without any complications. AFVwI was undetectable after 5 months with normalization of FV activity.

**Conclusion:**

Our case highlights the first pediatric case of thrombosis with a rare finding of AFVwI, successfully managed with anticoagulation therapy with complete resolution.

## Essentials

• AFVwI is a rare entity.
• This is the first pediatric case of acute deep venous thrombosis associated with the finding of AFVwI.
• Finding AFVwI can complicate anticoagulant management decisions.
• Anticoagulation with close clinical and laboratory monitoring is recommended.

## 1 Introduction

The development of acquired factor (F)V with inhibitor (AFVwI) is rare, with an estimated incidence of ∼0.09 to 0.29 cases/million that largely results in bleeding complications with sporadic case reports of thrombosis [[1], [2], [3], [4], [5]]. Etiologies known to be associated with the development of AFVwI include exposure to bovine thrombin, beta-lactam antibiotics, and underlying conditions such as malignancy and autoimmune diseases, with 20% of reported cases categorized as idiopathic. We describe the first pediatric case of acute deep venous thrombosis (DVT) associated with the concurrent finding of AFVwI, which impacted management decisions.

## 2 Case Report

A 13-year-old African American female presented with acute extensive DVT of the left lower extremity. Evaluation revealed a track participant, with a body mass index in the 20th percentile, dehydrated due to poor oral intake per the parental report, with a known underlying condition of Crohn disease, well controlled on therapy with 5 amino-salicylic acid without recent flares. The patient denied long-distance travel, use of estrogen-based hormonal therapy, recent antibiotic use, surgery, or family history of thrombosis. Ultrasound Doppler imaging revealed acute occlusive thrombus in the left superficial femoral, popliteal, gastrocnemius, anterior/posterior tibial, and peroneal veins. Computed tomography venography revealed evidence of May–Thurner anatomy with marked narrowing of the cephalad aspects of the left common iliac vein between the spine and the right common iliac artery. Prior to initiating anticoagulant therapy, a complete blood count revealed microcytic hypochromic iron deficiency anemia values notable for hemoglobin of 9 g/dL, ferritin at 15, and a platelet count of 430,000. Coagulation studies revealed a normal activated partial thromboplastin time (aPTT) of 31.6 seconds (normal range, 20.6-32.4 seconds) with an elevated FVIII activity of 332% (normal range, 53%-131%), fibrinogen activity of 516 mg/dL (normal range, 154-448 mg/dL), and a prolonged prothrombin time (PT)/INR of 17/1.7 (PT normal range, −8.8 to 11.1 seconds), which on repeat evaluation was 19.7/2 (see the Table; [6]). Despite 4 consecutive daily doses of intravenous vitamin K at 1 mg (days 1-4 of admission), the PT/INR remained persistently elevated at 19.7/2.0 on day 5. On days 2 and 3 of admission, mixing studies were performed, which failed to correct the PT, suggestive of an inhibitor. Testing was positive for a lupus anticoagulant (LA), including Sta-Clot (Stago), dilute Russell’s viper venom screen and ratio, and aPTT-LA (see the Table). On days 2 and 3, the patient was found to have a low FVII at 34% (repeat 28%) and 40%, respectively, which normalized to 68% by day 5 (normal range, 56%-115% for age); the patient had normal FII, FIX, and FX activity levels on day 2. The patient did demonstrate a low FV activity at 32% (repeat 21%), 22%, and 47% on days 2, 3, and 5, respectively (normal range, 55%-99% for age). Low FV persisted over 5 months (lowest, 21%; range, 21%-52%). FV antigen levels were measured over time by enzyme-linked immunosorbent assay and were within the normal range (10.3 ± 3.9 μg/mL; normal range, 7.0-10.0 μg/mL). Due to the identification of low FV activity in a patient with a negative bleeding history, AFVwI was considered, and testing was pursued. A FV inhibitor was detected by the Bethesda assay, and the highest titer was 12 Bethesda Units (BU), with a range of 4 to 12 BU over time. Five months after the initial diagnosis, AFVwI was undetectable, with a FV activity of 87%. LA (Sta-Clot) was negative in 4 weeks. D-dimer normalized on follow-up at 8 weeks to 0.22 mg/L fibrinogen equivalent units from an elevated value on admission of 4.7 mg/L fibrinogen equivalent units (cutoff 0.49). The elevated FVIII and fibrinogen were attributed to inflammation and contributed to the observed normal aPTT despite the low FV. Thrombophilia evaluation was negative, including normal antithrombin, protein C and S activity levels, and negative FV Leiden and prothrombin 20210 gene mutations; although for LA, the antiphospholipid antibody (anticardiolipin and anti-beta-2 glycoprotein) titers were negative.

**Table:** Pertinent laboratory values during the clinical course.

Hospital days of stay →	1	2 (LMWH started)	3	4	5	Follow-up mo →	1	3	>3-12
PT (8.8-11.7 s)	17/19.7^a^	22.4	20	19.9	19.7	ND			
INR	1.7/2.0^a^	2.28	2.03	2.03	2.00				
Mixing study PT-patient	ND	22.3	21.0	ND	ND				
PT-normal pool		10.7	10.8						
PT-1:1 mix		15.6, not corrected	15.6, not corrected						
FV (55-99 for age)^b^		32/21^a^	22		47	ND		52	>80
FV inhibitor (BU)		ND	4		12			4	<1
FVII (56-115 for age)		34/28	40		68			63	>60
STA-CLOT		Positive	ND		ND	ND	Negative		
aPTT-LA (28.6-41.9 s)		69.5	ND						
DRVVT screen (<45 s)		78							
DRVVT ratio (<1.3)		1.5							
Fibrinogen (154-448 mg/dL)		516							
FII (61-104 for age)		92							
FVIII (53-131 for age)		232							
FIX (59-122 for age)		125							
FX (50-117 for age)		82							
FXI (50-97 for age)		74							

aPTT, activated partial thromboplastin time; BU, Bethesda Units; DRVVT, Dilute Russell's Viper Venom Test; FII/FIX/FV/FVII/FVIII/FX/FXI, factor II/IX/V/VII/VIII/X/XI; INR, international normalized ratio; LA, lupus anticoagulant; LMWH, low-molecular-weight heparin; ND, not done; PT, prothrombin time.

a Repeat values on the same day.

b Normal range for ages 11 to 16 years, all in %.

Anticoagulation therapy was initiated at diagnosis of extensive DVT with low-molecular-weight heparin (LMWH) twice daily at 1 mg/kg/dose with anti-Xa levels maintained in the range of 0.5 to 0.8 with close clinical observation for associated bleeding due to the low FV activity. Based on the patient’s age and weight, despite the noted severe iliac vein stenosis due to May–Thurner anatomy, stenting of the vessel was not feasible. Approximately 6 months after initiation of therapeutic anticoagulation with LMWH, ultrasound Doppler imaging revealed chronic nonocclusive thrombus within the popliteal vein and the upper calf veins. The patient was transitioned to rivaroxaban at 15 mg once daily and then later adjusted for weight to 20 mg once daily through shared decision-making with the patient/family and after discussion with gastroenterology and national experts. Despite concerns regarding absorption due to Crohn disease, rivaroxaban was considered the most appropriate option as the patient’s disease was limited to the sigmoid colon and rectum, and she was opposed to continuation of injectable LMWH. Currently, the patient remains on life-long anticoagulation, given her ongoing risk factors of May–Thurner and Crohn disease. The patient continues to remain stable without bleeding symptoms on the standard dose of rivaroxaban (20 mg daily) without reported heavy menstrual flow. One year after the initial thrombotic event, no sonographic evidence of DVT remains in the left lower extremity.

The patient and her mother provided verbal consent prior to writing this manuscript for submission. This report was submitted abiding with ethical principles.

## 3 Discussion and Conclusion

This is the first reported pediatric case of AFVwI diagnosed with acute thrombosis. AFVwI contributing to thrombosis has mostly been observed in the elderly population (>60 years) [[2], [3], [4], [5]]. Our patient presented with multiple factors contributing to thrombosis, including possible dehydration, Crohn disease, May–Thurner anatomy, LA, and AFVwI. The rare finding of AFVwI with decreased FV activity and its reported association with both a hemorrhagic and thrombotic phenotype had important clinical implications for the patient’s acute and long-term management. AFVwI is most commonly an immunoglobulin (Ig)G antibody often directed to the second C-type domain of the light chain of FV, which is the binding site for phospholipids. AFVwI can result in a prothrombotic state through the inhibition of inactivation of FVa, inducing activated protein C (APC) resistance. AFVwI may also alter FV’s anticoagulant function in its role as a cofactor for tissue factor pathway inhibitor of FXa and its APC cofactor function in the inactivation of FVIIIa [7,8]. In our reported case, FV activity was reduced to the lowest value of 21%, with AFVwI as high as 12 BU during a 5-month course, indicating a partially neutralizing inhibitor. Positive LA, even in the absence of antiphospholipid antibody, is known to be a risk factor for thrombosis and has been described as coexistent with AFVwI, both resolving at the same time [5]. In our case, the patient was positive both for LA and for a specific FV inhibitor for the initial 4 weeks; however, the detection of the AFVwI persisted for up to 5 months despite early resolution of the LA, clinically supporting the concept of a specific FV inhibitor as opposed to nonspecific inhibitor interference in the assay. In certain cases, it may be difficult to discern an LA from a specific factor inhibitor when they coexist. Persons with hemophilia A can demonstrate false-positive FVIII inhibitor results related to LA, which are only distinguishable from clinically relevant FVIII inhibitors by fluorescence immunoassay [9]. The transiently decreased FVII was attributed to the LA, which can partially or completely neutralize specific factors and possible vitamin K deficiency since FVII levels were corrected postreplacement.

To elucidate the properties of the AFVwI, total IgG from 1 mL of plasma from day 3 of hospitalization when the FV inhibitor titer was 4 BU was purified. Total IgG was also purified from normal plasma, serving as the control. Using biolayer interferometry, the patient’s IgG bound to FV was immobilized to the sensor tip (Figure A). This was comparable with a control FV antibody, while IgG purified from normal plasma did not bind (data not shown). We also detected binding to FV in this system using the patient’s plasma from day 3 of hospitalization (data not shown). In a thrombin generation assay, the patient’s IgG prolonged the lag time when added to normal plasma, consistent with the prolonged PT (Figure B). Importantly, the patient’s IgG increased both peak thrombin and endogenous thrombin potential, suggesting that the antibody enhanced coagulation when bound to FV (Figure B). Similar results were seen with patient IgG using FV-deficient plasma with added plasma-derived or recombinant FV (not shown) but not with FVa (Figure C). These data suggest that the patient’s IgG may alter FV’s anticoagulant function, as FVa does not exhibit this activity [10,11]. In support of this hypothesis, we found that the patient’s IgG partially restored thrombin generation in the presence of APC, where control IgG has no effect (Figure D). Due to limited amounts of patient IgG and the low FV inhibitor titer, we were unable to discern a precise mechanism of action. However, the data suggest that the patient’s FV antibody was able to enhance coagulation and may induce partial APC resistance or alter FV’s anticoagulant cofactor function for APC or tissue factor pathway inhibitor.

**Figure:**
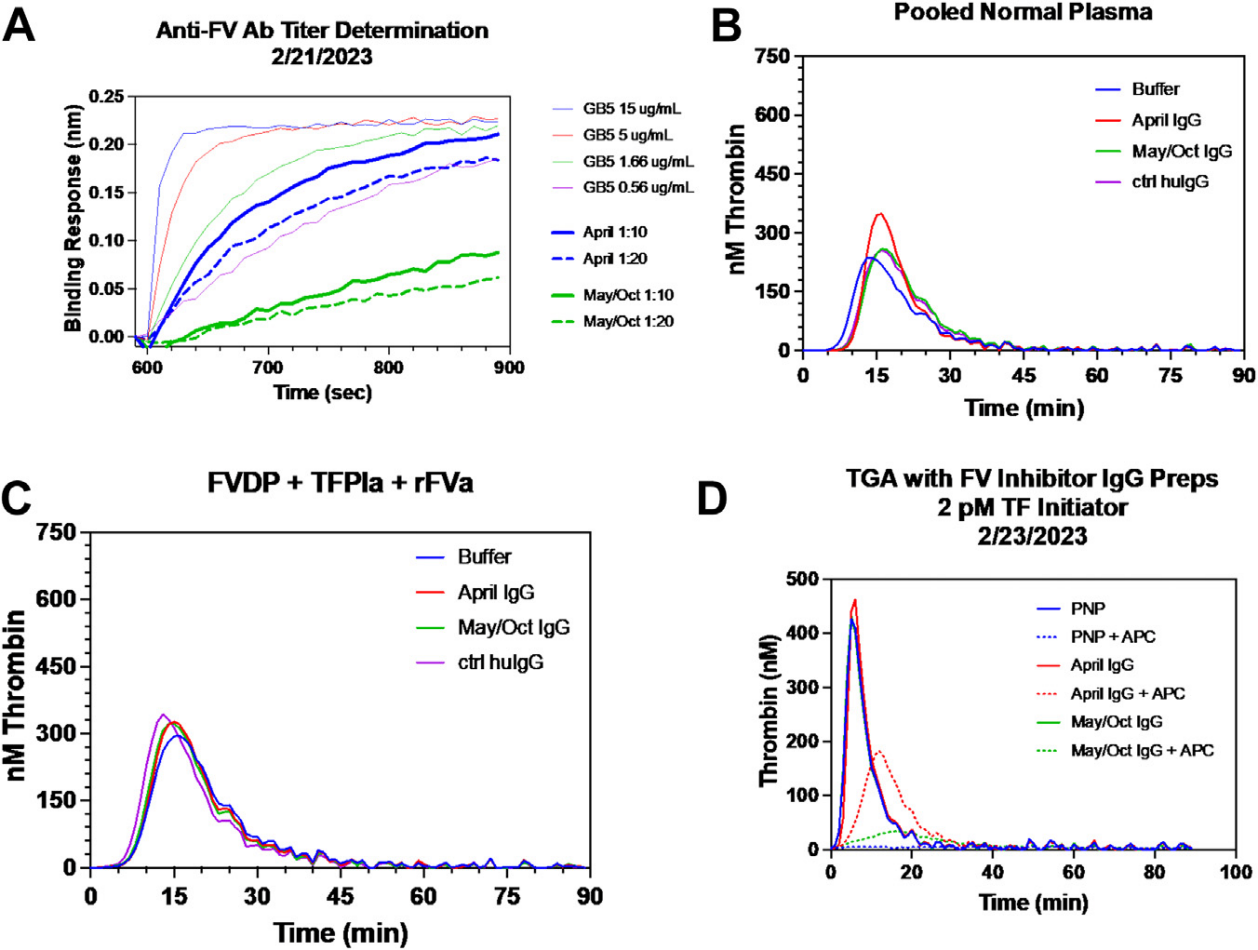
An acquired antifactor (F)V (aFV) antibody enhances thrombin generation (TG) in plasma. (A) Binding of total immunoglobulin (Ig)G purified from patient plasma to FV. Biotin-labeled FV was immobilized on streptavidin biosensors, and the binding response of purified total IgG from the patient to the immobilized FV was measured. Several concentrations of a previously characterized FV-binding monoclonal antibody were also measured as a control (ctrl). (B) TG in pooled normal plasma (PNP). Patient total IgG, ctrl human IgG (huIgG), or assay buffer were diluted into PNP (5:1 PNP:IgG), and TG was initiated with 0.1 pM tissue factor. (C) TG in FV-deficient plasma reconstituted with 2 nM recombinant FVa was measured in the presence of buffer, patient IgG, or ctrl IgG, as described in (B). (D) TG in PNP treated with activated protein C (APC). Patient total IgG or buffer was diluted into PNP (5:1 PNP:IgG), and TG was initiated with 2 pM tissue factor immediately after adding 6 nM APC.

Adult cases of AFVwI and venous thrombosis have been reported in the literature and summarized by Ghachem et al. [3]. The age range was from 44 to 82 years, associated with sepsis, antibiotics, and malignancy, with the FV inhibitor ranging from ∼3 BU to 160 BU, remission achieved in 2 weeks to 6 months with rare cases warranting steroids.

Discussions were held with gastroenterologists regarding steroid use or disease-modifying therapeutics for the underlying Crohn disease, which could also impact the AFVwI as a secondary outcome; these modalities were not initiated due to the stable course of the patient’s thrombosis and stable underlying Crohn disease. Immune modifiers could be reasonable therapeutic options in similar clinical scenarios [[1], [2], [3], [4], [5]].

This case highlights a rare finding of AFVwI in a pediatric case of thrombosis with an underlying chronic inflammatory autoimmune disorder. Initial detailed evaluation and close clinical follow-up until resolution with careful consideration of options is required in these rare circumstances. The potential risks and benefits of interventions must be weighed carefully when encountering complicated clinical hemostatic conditions, as exemplified by this case.
